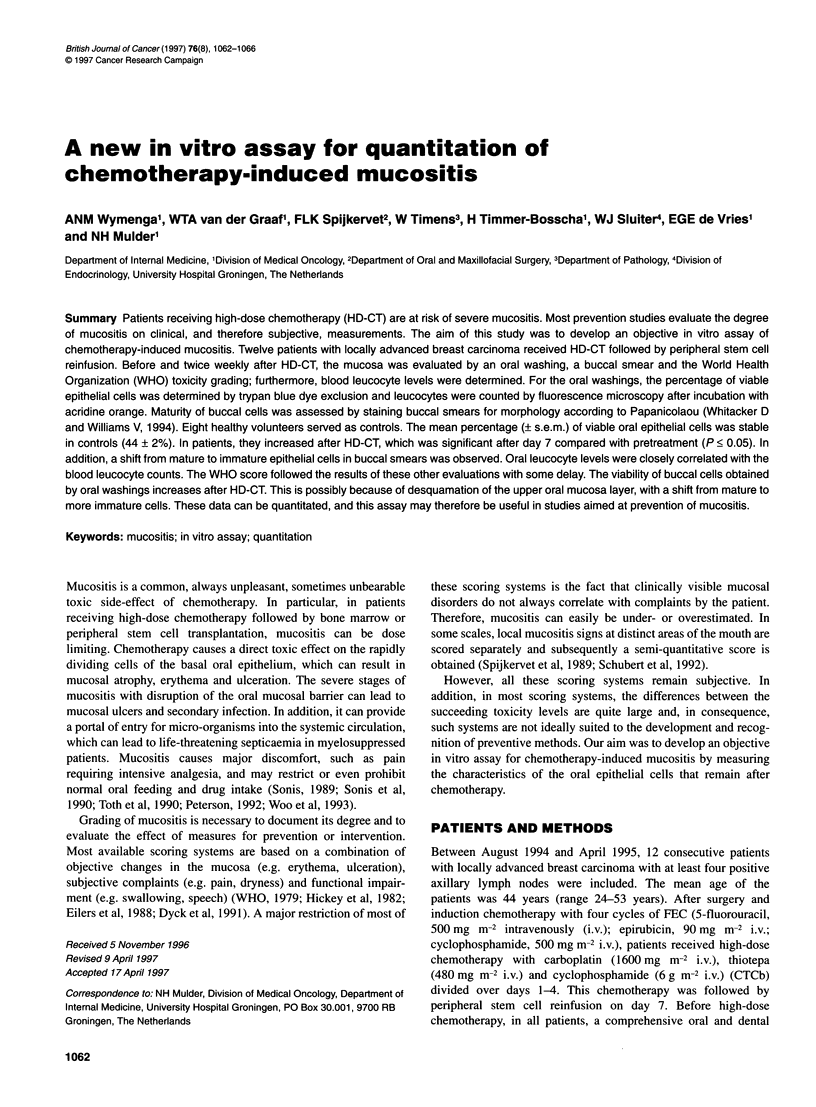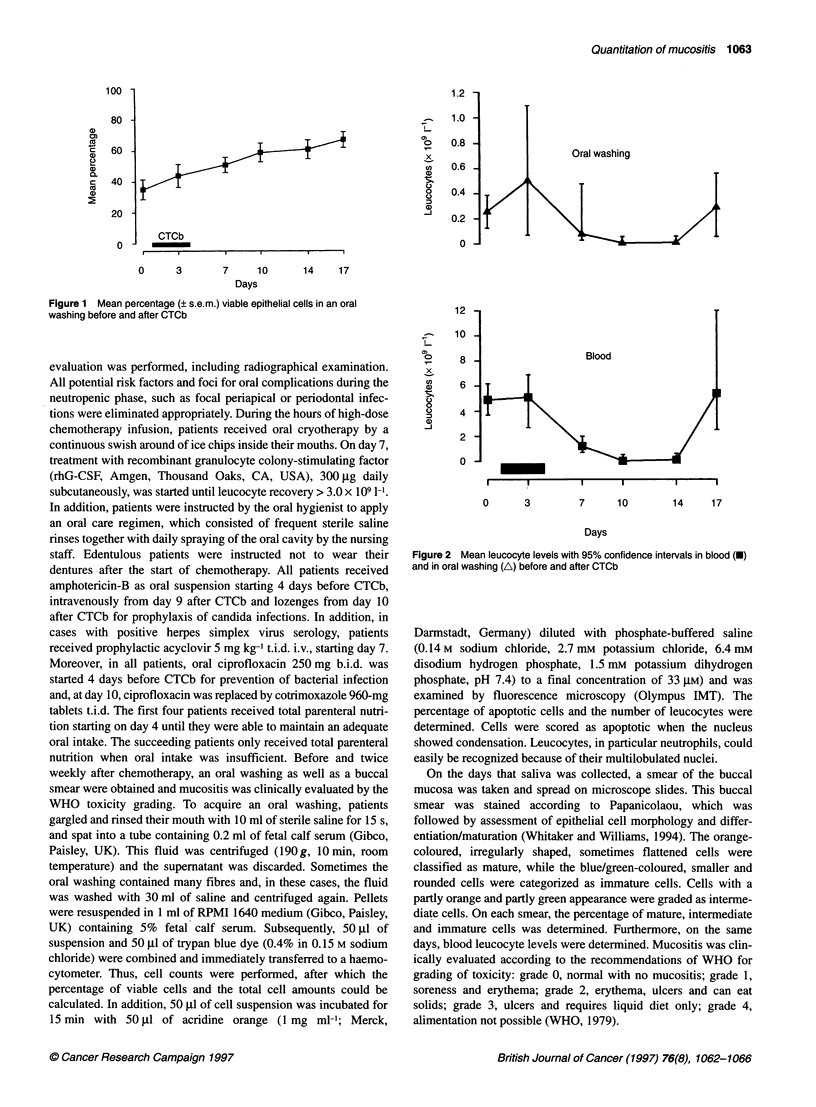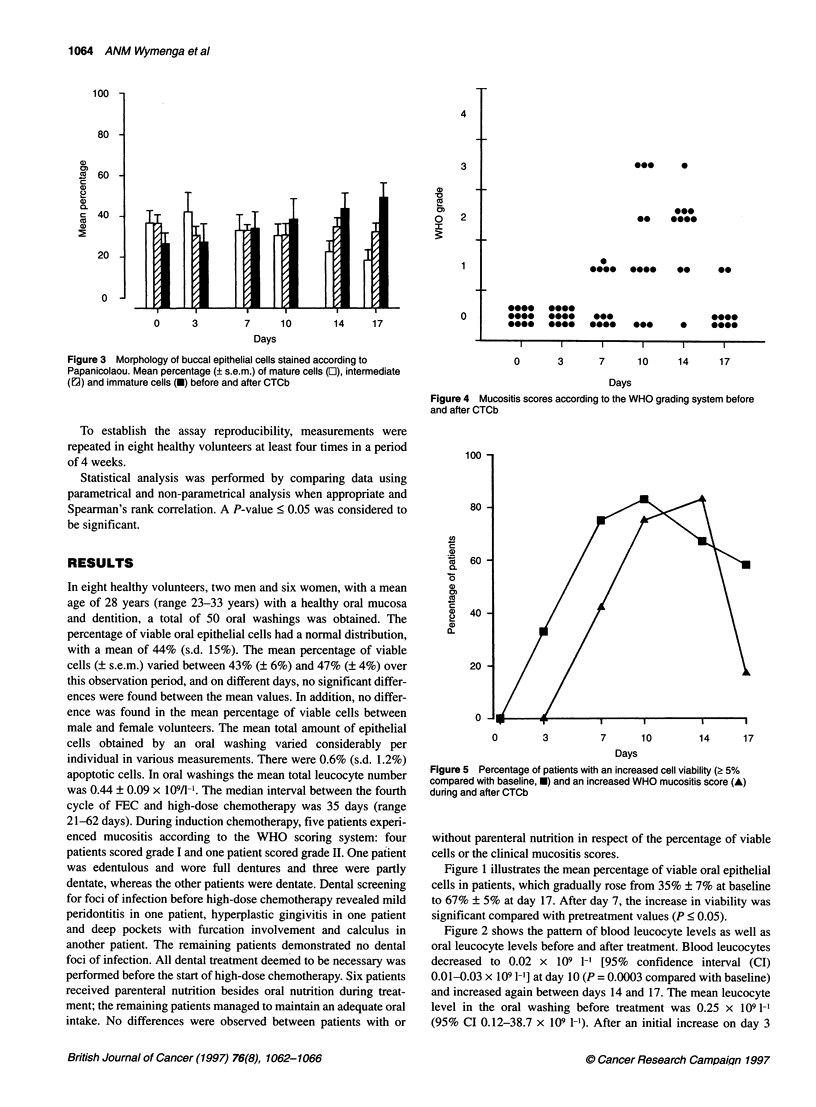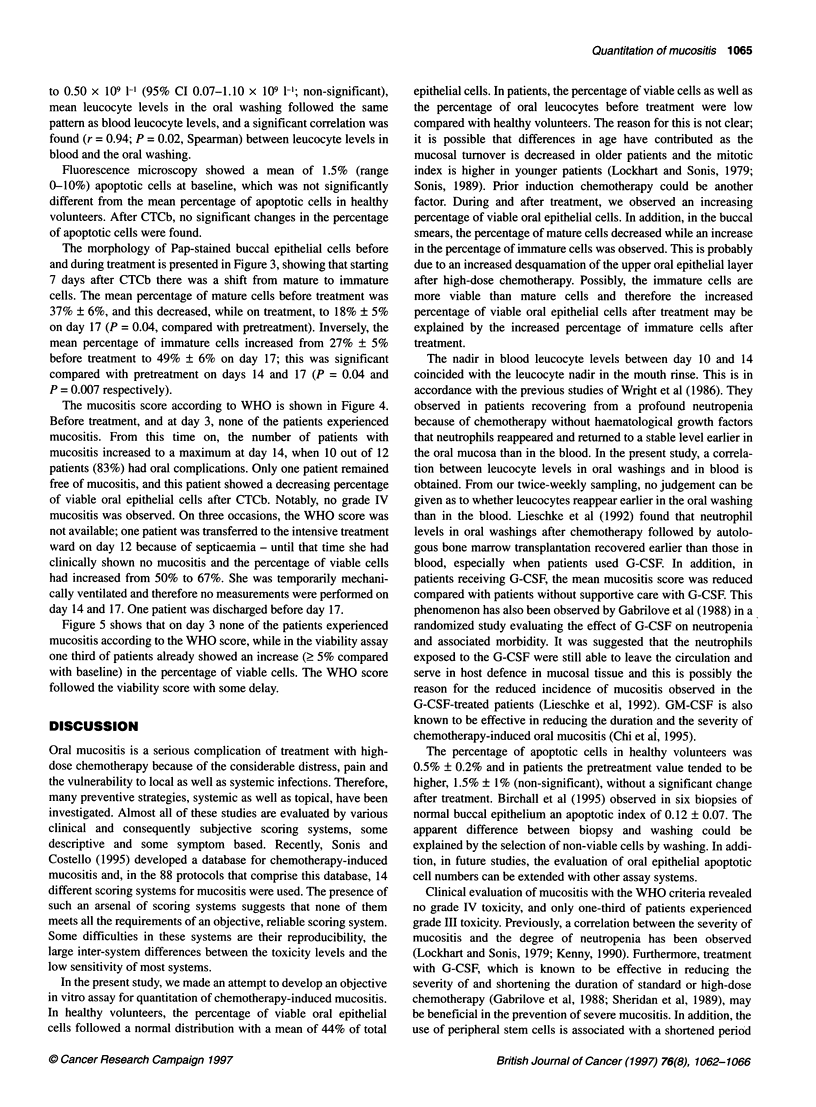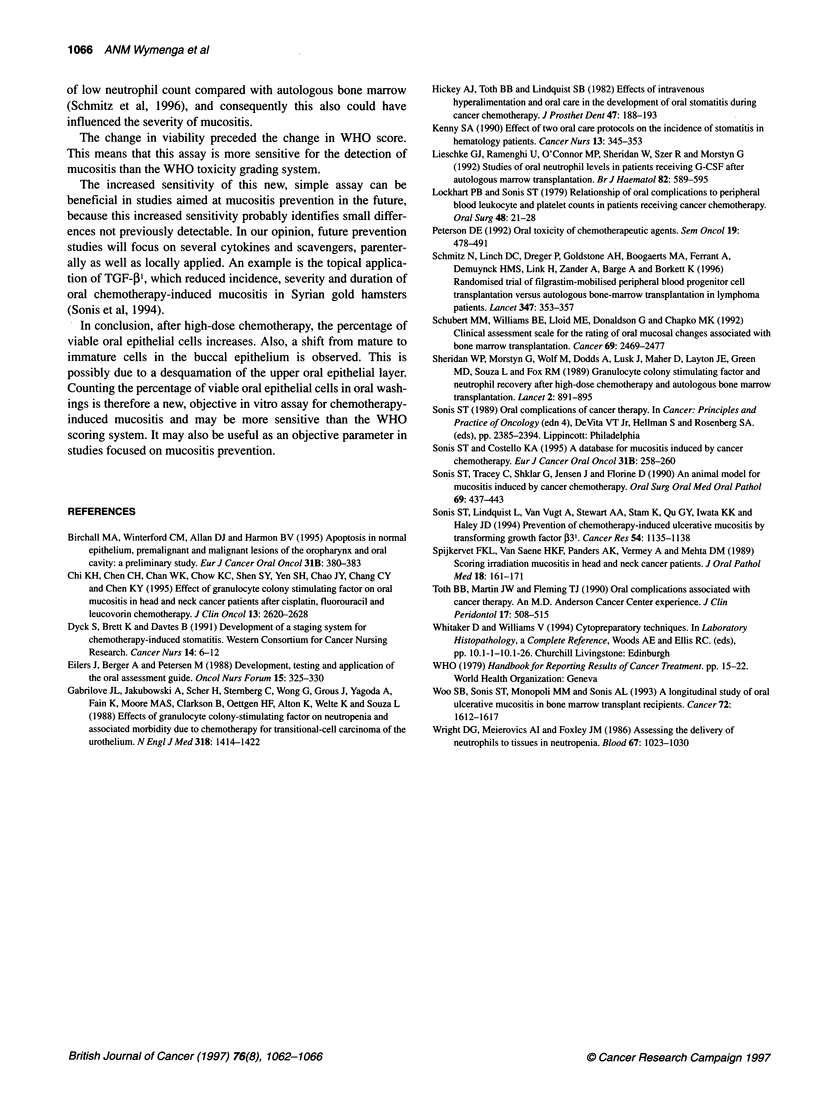# A new in vitro assay for quantitation of chemotherapy-induced mucositis.

**DOI:** 10.1038/bjc.1997.508

**Published:** 1997

**Authors:** A. N. Wymenga, W. T. van der Graaf, F. L. Spijkervet, W. Timens, H. Timmer-Bosscha, W. J. Sluiter, E. G. de Vries, N. H. Mulder

**Affiliations:** Department of Internal Medicine, University Hospital Groningen, The Netherlands.

## Abstract

Patients receiving high-dose chemotherapy (HD-CT) are at risk of severe mucositis. Most prevention studies evaluate the degree of mucositis on clinical, and therefore subjective, measurements. The aim of this study was to develop an objective in vitro assay of chemotherapy-induced mucositis. Twelve patients with locally advanced breast carcinoma received HD-CT followed by peripheral stem cell reinfusion. Before and twice weekly after HD-CT, the mucosa was evaluated by an oral washing, a buccal smear and the World Health Organization (WHO) toxicity grading; furthermore, blood leucocyte levels were determined. For the oral washings, the percentage of viable epithelial cells was determined by trypan blue dye exclusion and leucocytes were counted by fluorescence microscopy after incubation with acridine orange. Maturity of buccal cells was assessed by staining buccal smears for morphology according to Papanicolaou (Whitacker D and Williams V, 1994). Eight healthy volunteers served as controls. The mean percentage (+/- s.e.m.) of viable oral epithelial cells was stable in controls (44 +/- 2%). In patients, they increased after HD-CT, which was significant after day 7 compared with pretreatment (P < or = 0.05). In addition, a shift from mature to immature epithelial cells in buccal smears was observed. Oral leucocyte levels were closely correlated with the blood leucocyte counts. The WHO score followed the results of these other evaluations with some delay. The viability of buccal cells obtained by oral washings increases after HD-CT. This is possibly because of desquamation of the upper oral mucosa layer, with a shift from mature to more immature cells. These data can be quantitated, and this assay may therefore be useful in studies aimed at prevention of mucositis.


*British Journal of Cancer* (1997) **76**(8), 1062–1066
© 1997 Cancer Research Campaign

### A new in vitro assay for quantitation of chemotherapy-induced mucositis

**ANM Wymenga[1], WTA van der Graaf[1], FLK Spijkervet[2], W Timens[3], H Timmer-Bosscha[1], WJ Sluiter[4], EGE de Vries[1] and NH Mulder[1]**

Department of Internal Medicine, [1]Division of Medical Oncology, [2]Department of Oral and Maxillofacial Surgery, [3]Department of Pathology, [4]Division of Endocrinology, University Hospital Groningen, The Netherlands

**Summary** Patients receiving high-dose chemotherapy (HD-CT) are at risk of severe mucositis. Most prevention studies evaluate the degree of mucositis on clinical, and therefore subjective, measurements. The aim of this study was to develop an objective in vitro assay of chemotherapy-induced mucositis. Twelve patients with locally advanced breast carcinoma received HD-CT followed by peripheral stem cell reinfusion. Before and twice weekly after HD-CT, the mucosa was evaluated by an oral washing, a buccal smear and the World Health Organization (WHO) toxicity grading; furthermore, blood leucocyte levels were determined. For the oral washings, the percentage of viable epithelial cells was determined by trypan blue dye exclusion and leucocytes were counted by fluorescence microscopy after incubation with acridine orange. Maturity of buccal cells was assessed by staining buccal smears for morphology according to Papanicolaou (Whitacker D and Williams V, 1994). Eight healthy volunteers served as controls. The mean percentage (± s.e.m.) of viable oral epithelial cells was stable in controls (44 ± 2%). In patients, they increased after HD-CT, which was significant after day 7 compared with pretreatment ($P \leq 0.05$). In addition, a shift from mature to immature epithelial cells in buccal smears was observed. Oral leucocyte levels were closely correlated with the blood leucocyte counts. The WHO score followed the results of these other evaluations with some delay. The viability of buccal cells obtained by oral washings increases after HD-CT. This is possibly because of desquamation of the upper oral mucosa layer, with a shift from mature to more immature cells. These data can be quantitated, and this assay may therefore be useful in studies aimed at prevention of mucositis.

**Keywords:** mucositis; in vitro assay; quantitation

Mucositis is a common, always unpleasant, sometimes unbearable toxic side-effect of chemotherapy. In particular, in patients receiving high-dose chemotherapy followed by bone marrow or peripheral stem cell transplantation, mucositis can be dose limiting. Chemotherapy causes a direct toxic effect on the rapidly dividing cells of the basal oral epithelium, which can result in mucosal atrophy, erythema and ulceration. The severe stages of mucositis with disruption of the oral mucosal barrier can lead to mucosal ulcers and secondary infection. In addition, it can provide a portal of entry for micro-organisms into the systemic circulation, which can lead to life-threatening septicaemia in myelosuppressed patients. Mucositis causes major discomfort, such as pain requiring intensive analgesia, and may restrict or even prohibit normal oral feeding and drug intake (Sonis, 1989; Sonis et al, 1990; Toth et al, 1990; Peterson, 1992; Woo et al, 1993).

Grading of mucositis is necessary to document its degree and to evaluate the effect of measures for prevention or intervention. Most available scoring systems are based on a combination of objective changes in the mucosa (e.g. erythema, ulceration), subjective complaints (e.g. pain, dryness) and functional impairment (e.g. swallowing, speech) (WHO, 1979; Hickey et al, 1982; Eilers et al, 1988; Dyck et al, 1991). A major restriction of most of these scoring systems is the fact that clinically visible mucosal disorders do not always correlate with complaints by the patient. Therefore, mucositis can easily be under- or overestimated. In some scales, local mucositis signs at distinct areas of the mouth are scored separately and subsequently a semi-quantitative score is obtained (Spijkervet et al, 1989; Schubert et al, 1992).

However, all these scoring systems remain subjective. In addition, in most scoring systems, the differences between the succeeding toxicity levels are quite large and, in consequence, such systems are not ideally suited to the development and recognition of preventive methods. Our aim was to develop an objective in vitro assay for chemotherapy-induced mucositis by measuring the characteristics of the oral epithelial cells that remain after chemotherapy.

*Received 5 November 1996*
*Revised 9 April 1997*
*Accepted 17 April 1997*

*Correspondence to:* NH Mulder, Division of Medical Oncology, Department of Internal Medicine, University Hospital Groningen, PO Box 30.001, 9700 RB Groningen, The Netherlands

### PATIENTS AND METHODS

Between August 1994 and April 1995, 12 consecutive patients with locally advanced breast carcinoma with at least four positive axillary lymph nodes were included. The mean age of the patients was 44 years (range 24–53 years). After surgery and induction chemotherapy with four cycles of FEC (5-fluorouracil, 500 mg $m^{-2}$ intravenously (i.v.); epirubicin, 90 mg $m^{-2}$ i.v.; cyclophosphamide, 500 mg $m^{-2}$ i.v.), patients received high-dose chemotherapy with carboplatin (1600 mg $m^{-2}$ i.v.), thiotepa (480 mg $m^{-2}$ i.v.) and cyclophosphamide (6 g $m^{-2}$ i.v.) (CTCb) divided over days 1–4. This chemotherapy was followed by peripheral stem cell reinfusion on day 7. Before high-dose chemotherapy, in all patients, a comprehensive oral and dental

**Figure 1** Mean percentage (± s.e.m.) viable epithelial cells in an oral washing before and after CTCb

**Figure 2** Mean leucocyte levels with 95% confidence intervals in blood (■) and in oral washing (△) before and after CTCb evaluation was performed, including radiographical examination. All potential risk factors and foci for oral complications during the neutropenic phase, such as focal periapical or periodontal infections were eliminated appropriately. During the hours of high-dose chemotherapy infusion, patients received oral cryotherapy by a continuous swish around of ice chips inside their mouths. On day 7, treatment with recombinant granulocyte colony-stimulating factor (rhG-CSF, Amgen, Thousand Oaks, CA, USA), 300 μg daily subcutaneously, was started until leucocyte recovery > $3.0 \times 10^9$ $l^{-1}$. In addition, patients were instructed by the oral hygienist to apply an oral care regimen, which consisted of frequent sterile saline rinses together with daily spraying of the oral cavity by the nursing staff. Edentulous patients were instructed not to wear their dentures after the start of chemotherapy. All patients received amphotericin-B as oral suspension starting 4 days before CTCb, intravenously from day 9 after CTCb and lozenges from day 10 after CTCb for prophylaxis of candida infections. In addition, in cases with positive herpes simplex virus serology, patients received prophylactic acyclovir 5 mg $kg^{-1}$ t.i.d. i.v., starting day 7. Moreover, in all patients, oral ciprofloxacin 250 mg b.i.d. was started 4 days before CTCb for prevention of bacterial infection and, at day 10, ciprofloxacin was replaced by cotrimoxazole 960-mg tablets t.i.d. The first four patients received total parenteral nutrition starting on day 4 until they were able to maintain an adequate oral intake. The succeeding patients only received total parenteral nutrition when oral intake was insufficient. Before and twice weekly after chemotherapy, an oral washing as well as a buccal smear were obtained and mucositis was clinically evaluated by the WHO toxicity grading. To acquire an oral washing, patients gargled and rinsed their mouth with 10 ml of sterile saline for 15 s, and spat into a tube containing 0.2 ml of fetal calf serum (Gibco, Paisley, UK). This fluid was centrifuged (190 *g*, 10 min, room temperature) and the supernatant was discarded. Sometimes the oral washing contained many fibres and, in these cases, the fluid was washed with 30 ml of saline and centrifuged again. Pellets were resuspended in 1 ml of RPMI 1640 medium (Gibco, Paisley, UK) containing 5% fetal calf serum. Subsequently, 50 μl of suspension and 50 μl of trypan blue dye (0.4% in 0.15 M sodium chloride) were combined and immediately transferred to a haemocytometer. Thus, cell counts were performed, after which the percentage of viable cells and the total cell amounts could be calculated. In addition, 50 μl of cell suspension was incubated for 15 min with 50 μl of acridine orange (1 mg $ml^{-1}$; Merck, Darmstadt, Germany) diluted with phosphate-buffered saline (0.14 M sodium chloride, 2.7 mM potassium chloride, 6.4 mM disodium hydrogen phosphate, 1.5 mM potassium dihydrogen phosphate, pH 7.4) to a final concentration of 33 μM) and was examined by fluorescence microscopy (Olympus IMT). The percentage of apoptotic cells and the number of leucocytes were determined. Cells were scored as apoptotic when the nucleus showed condensation. Leucocytes, in particular neutrophils, could easily be recognized because of their multilobulated nuclei.

On the days that saliva was collected, a smear of the buccal mucosa was taken and spread on microscope slides. This buccal smear was stained according to Papanicolaou, which was followed by assessment of epithelial cell morphology and differentiation/maturation (Whitaker and Williams, 1994). The orange-coloured, irregularly shaped, sometimes flattened cells were classified as mature, while the blue/green-coloured, smaller and rounded cells were categorized as immature cells. Cells with a partly orange and partly green appearance were graded as intermediate cells. On each smear, the percentage of mature, intermediate and immature cells was determined. Furthermore, on the same days, blood leucocyte levels were determined. Mucositis was clinically evaluated according to the recommendations of WHO for grading of toxicity: grade 0, normal with no mucositis; grade 1, soreness and erythema; grade 2, erythema, ulcers and can eat solids; grade 3, ulcers and requires liquid diet only; grade 4, alimentation not possible (WHO, 1979).

*© Cancer Research Campaign 1997*

Figure 3 Morphology of buccal epithelial cells stained according to Papanicolaou. Mean percentage (± s.e.m.) of mature cells (□), intermediate (▨) and immature cells (■) before and after CTCb

Figure 4 Mucositis scores according to the WHO grading system before and after CTCb

To establish the assay reproducibility, measurements were repeated in eight healthy volunteers at least four times in a period of 4 weeks.

Statistical analysis was performed by comparing data using parametrical and non-parametrical analysis when appropriate and Spearman's rank correlation. A $P$-value $\leq 0.05$ was considered to be significant.

### RESULTS

In eight healthy volunteers, two men and six women, with a mean age of 28 years (range 23–33 years) with a healthy oral mucosa and dentition, a total of 50 oral washings was obtained. The percentage of viable oral epithelial cells had a normal distribution, with a mean of 44% (s.d. 15%). The mean percentage of viable cells (± s.e.m.) varied between 43% (± 6%) and 47% (± 4%) over this observation period, and on different days, no significant differences were found between the mean values. In addition, no difference was found in the mean percentage of viable cells between male and female volunteers. The mean total amount of epithelial cells obtained by an oral washing varied considerably per individual in various measurements. There were 0.6% (s.d. 1.2%) apoptotic cells. In oral washings the mean total leucocyte number was $0.44 \pm 0.09 \times 10^9/l^{-1}$. The median interval between the fourth cycle of FEC and high-dose chemotherapy was 35 days (range 21–62 days). During induction chemotherapy, five patients experienced mucositis according to the WHO scoring system: four patients scored grade I and one patient scored grade II. One patient was edentulous and wore full dentures and three were partly dentate, whereas the other patients were dentate. Dental screening for foci of infection before high-dose chemotherapy revealed mild peridontitis in one patient, hyperplastic gingivitis in one patient and deep pockets with furcation involvement and calculus in another patient. The remaining patients demonstrated no dental foci of infection. All dental treatment deemed to be necessary was performed before the start of high-dose chemotherapy. Six patients received parenteral nutrition besides oral nutrition during treatment; the remaining patients managed to maintain an adequate oral intake. No differences were observed between patients with or

Figure 5 Percentage of patients with an increased cell viability (≥ 5% compared with baseline, ■) and an increased WHO mucositis score (▲) during and after CTCb without parenteral nutrition in respect of the percentage of viable cells or the clinical mucositis scores.

Figure 1 illustrates the mean percentage of viable oral epithelial cells in patients, which gradually rose from 35% ± 7% at baseline to 67% ± 5% at day 17. After day 7, the increase in viability was significant compared with pretreatment values ($P \leq 0.05$).

Figure 2 shows the pattern of blood leucocyte levels as well as oral leucocyte levels before and after treatment. Blood leucocytes decreased to $0.02 \times 10^9$ $l^{-1}$ [95% confidence interval (CI) $0.01–0.03 \times 10^9$ $l^{-1}$] at day 10 ($P = 0.0003$ compared with baseline) and increased again between days 14 and 17. The mean leucocyte level in the oral washing before treatment was $0.25 \times 10^9$ $l^{-1}$ (95% CI $0.12–38.7 \times 10^9$ $l^{-1}$). After an initial increase on day 3

© Cancer Research Campaign 1997

to $0.50 \times 10^9\ l^{-1}$ (95% CI 0.07–1.10 $\times 10^9\ l^{-1}$; non-significant), mean leucocyte levels in the oral washing followed the same pattern as blood leucocyte levels, and a significant correlation was found ($r = 0.94$; $P = 0.02$, Spearman) between leucocyte levels in blood and the oral washing.

Fluorescence microscopy showed a mean of 1.5% (range 0–10%) apoptotic cells at baseline, which was not significantly different from the mean percentage of apoptotic cells in healthy volunteers. After CTCb, no significant changes in the percentage of apoptotic cells were found.

The morphology of Pap-stained buccal epithelial cells before and during treatment is presented in Figure 3, showing that starting 7 days after CTCb there was a shift from mature to immature cells. The mean percentage of mature cells before treatment was 37% ± 6%, and this decreased, while on treatment, to 18% ± 5% on day 17 ($P = 0.04$, compared with pretreatment). Inversely, the mean percentage of immature cells increased from 27% ± 5% before treatment to 49% ± 6% on day 17; this was significant compared with pretreatment on days 14 and 17 ($P = 0.04$ and $P = 0.007$ respectively).

The mucositis score according to WHO is shown in Figure 4. Before treatment, and at day 3, none of the patients experienced mucositis. From this time on, the number of patients with mucositis increased to a maximum at day 14, when 10 out of 12 patients (83%) had oral complications. Only one patient remained free of mucositis, and this patient showed a decreasing percentage of viable oral epithelial cells after CTCb. Notably, no grade IV mucositis was observed. On three occasions, the WHO score was not available; one patient was transferred to the intensive treatment ward on day 12 because of septicaemia – until that time she had clinically shown no mucositis and the percentage of viable cells had increased from 50% to 67%. She was temporarily mechanically ventilated and therefore no measurements were performed on day 14 and 17. One patient was discharged before day 17.

Figure 5 shows that on day 3 none of the patients experienced mucositis according to the WHO score, while in the viability assay one third of patients already showed an increase (≥ 5% compared with baseline) in the percentage of viable cells. The WHO score followed the viability score with some delay.

### DISCUSSION

Oral mucositis is a serious complication of treatment with high-dose chemotherapy because of the considerable distress, pain and the vulnerability to local as well as systemic infections. Therefore, many preventive strategies, systemic as well as topical, have been investigated. Almost all of these studies are evaluated by various clinical and consequently subjective scoring systems, some descriptive and some symptom based. Recently, Sonis and Costello (1995) developed a database for chemotherapy-induced mucositis and, in the 88 protocols that comprise this database, 14 different scoring systems for mucositis were used. The presence of such an arsenal of scoring systems suggests that none of them meets all the requirements of an objective, reliable scoring system. Some difficulties in these systems are their reproducibility, the large inter-system differences between the toxicity levels and the low sensitivity of most systems.

In the present study, we made an attempt to develop an objective in vitro assay for quantitation of chemotherapy-induced mucositis. In healthy volunteers, the percentage of viable oral epithelial cells followed a normal distribution with a mean of 44% of total epithelial cells. In patients, the percentage of viable cells as well as the percentage of oral leucocytes before treatment were low compared with healthy volunteers. The reason for this is not clear; it is possible that differences in age have contributed as the mucosal turnover is decreased in older patients and the mitotic index is higher in younger patients (Lockhart and Sonis, 1979; Sonis, 1989). Prior induction chemotherapy could be another factor. During and after treatment, we observed an increasing percentage of viable oral epithelial cells. In addition, in the buccal smears, the percentage of mature cells decreased while an increase in the percentage of immature cells was observed. This is probably due to an increased desquamation of the upper oral epithelial layer after high-dose chemotherapy. Possibly, the immature cells are more viable than mature cells and therefore the increased percentage of viable oral epithelial cells after treatment may be explained by the increased percentage of immature cells after treatment.

The nadir in blood leucocyte levels between day 10 and 14 coincided with the leucocyte nadir in the mouth rinse. This is in accordance with the previous studies of Wright et al (1986). They observed in patients recovering from a profound neutropenia because of chemotherapy without haematological growth factors that neutrophils reappeared and returned to a stable level earlier in the oral mucosa than in the blood. In the present study, a correlation between leucocyte levels in oral washings and in blood is obtained. From our twice-weekly sampling, no judgement can be given as to whether leucocytes reappear earlier in the oral washing than in the blood. Lieschke et al (1992) found that neutrophil levels in oral washings after chemotherapy followed by autologous bone marrow transplantation recovered earlier than those in blood, especially when patients used G-CSF. In addition, in patients receiving G-CSF, the mean mucositis score was reduced compared with patients without supportive care with G-CSF. This phenomenon has also been observed by Gabrilove et al (1988) in a randomized study evaluating the effect of G-CSF on neutropenia and associated morbidity. It was suggested that the neutrophils exposed to the G-CSF were still able to leave the circulation and serve in host defence in mucosal tissue and this is possibly the reason for the reduced incidence of mucositis observed in the G-CSF-treated patients (Lieschke et al, 1992). GM-CSF is also known to be effective in reducing the duration and the severity of chemotherapy-induced oral mucositis (Chi et al, 1995).

The percentage of apoptotic cells in healthy volunteers was 0.5% ± 0.2% and in patients the pretreatment value tended to be higher, 1.5% ± 1% (non-significant), without a significant change after treatment. Birchall et al (1995) observed in six biopsies of normal buccal epithelium an apoptotic index of 0.12 ± 0.07. The apparent difference between biopsy and washing could be explained by the selection of non-viable cells by washing. In addition, in future studies, the evaluation of oral epithelial apoptotic cell numbers can be extended with other assay systems.

Clinical evaluation of mucositis with the WHO criteria revealed no grade IV toxicity, and only one-third of patients experienced grade III toxicity. Previously, a correlation between the severity of mucositis and the degree of neutropenia has been observed (Lockhart and Sonis, 1979; Kenny, 1990). Furthermore, treatment with G-CSF, which is known to be effective in reducing the severity of and shortening the duration of standard or high-dose chemotherapy (Gabrilove et al, 1988; Sheridan et al, 1989), may be beneficial in the prevention of severe mucositis. In addition, the use of peripheral stem cells is associated with a shortened period

© Cancer Research Campaign 1997

of low neutrophil count compared with autologous bone marrow (Schmitz et al, 1996), and consequently this also could have influenced the severity of mucositis.

The change in viability preceded the change in WHO score. This means that this assay is more sensitive for the detection of mucositis than the WHO toxicity grading system.

The increased sensitivity of this new, simple assay can be beneficial in studies aimed at mucositis prevention in the future, because this increased sensitivity probably identifies small differences not previously detectable. In our opinion, future prevention studies will focus on several cytokines and scavengers, parenterally as well as locally applied. An example is the topical application of TGF-$\beta^1$, which reduced incidence, severity and duration of oral chemotherapy-induced mucositis in Syrian gold hamsters (Sonis et al, 1994).

In conclusion, after high-dose chemotherapy, the percentage of viable oral epithelial cells increases. Also, a shift from mature to immature cells in the buccal epithelium is observed. This is possibly due to a desquamation of the upper oral epithelial layer. Counting the percentage of viable oral epithelial cells in oral washings is therefore a new, objective in vitro assay for chemotherapy-induced mucositis and may be more sensitive than the WHO scoring system. It may also be useful as an objective parameter in studies focused on mucositis prevention.

### REFERENCES

Birchall MA, Winterford CM, Allan DJ and Harmon BV (1995) Apoptosis in normal epithelium, premalignant and malignant lesions of the oropharynx and oral cavity: a preliminary study. *Eur J Cancer Oral Oncol* **31B**: 380–383

Chi KH, Chen CH, Chan WK, Chow KC, Shen SY, Yen SH, Chao JY, Chang CY and Chen KY (1995) Effect of granulocyte colony stimulating factor on oral mucositis in head and neck cancer patients after cisplatin, fluorouracil and leucovorin chemotherapy. *J Clin Oncol* **13**: 2620–2628

Dyck S, Brett K and Davtes B (1991) Development of a staging system for chemotherapy-induced stomatitis. Western Consortium for Cancer Nursing Research. *Cancer Nurs* **14**: 6–12

Eilers J, Berger A and Petersen M (1988) Development, testing and application of the oral assessment guide. *Oncol Nurs Forum* **15**: 325–330

Gabrilove JL, Jakubowski A, Scher H, Sternberg C, Wong G, Grous J, Yagoda A, Fain K, Moore MAS, Clarkson B, Oettgen HF, Alton K, Welte K and Souza L (1988) Effects of granulocyte colony-stimulating factor on neutropenia and associated morbidity due to chemotherapy for transitional-cell carcinoma of the urothelium. *N Engl J Med* **318**: 1414–1422

Hickey AJ, Toth BB and Lindquist SB (1982) Effects of intravenous hyperalimentation and oral care in the development of oral stomatitis during cancer chemotherapy. *J Prosthet Dent* **47**: 188–193

Kenny SA (1990) Effect of two oral care protocols on the incidence of stomatitis in hematology patients. *Cancer Nurs* **13**: 345–353

Lieschke GJ, Ramenghi U, O'Connor MP, Sheridan W, Szer R and Morstyn G (1992) Studies of oral neutrophil levels in patients receiving G-CSF after autologous marrow transplantation. *Br J Haematol* **82**: 589–595

Lockhart PB and Sonis ST (1979) Relationship of oral complications to peripheral blood leukocyte and platelet counts in patients receiving cancer chemotherapy. *Oral Surg* **48**: 21–28

Peterson DE (1992) Oral toxicity of chemotherapeutic agents. *Sem Oncol* **19**: 478–491

Schmitz N, Linch DC, Dreger P, Goldstone AH, Boogaerts MA, Ferrant A, Demuynck HMS, Link H, Zander A, Barge A and Borkett K (1996) Randomised trial of filgrastim-mobilised peripheral blood progenitor cell transplantation versus autologous bone-marrow transplantation in lymphoma patients. *Lancet* **347**: 353–357

Schubert MM, Williams BE, Lloid ME, Donaldson G and Chapko MK (1992) Clinical assessment scale for the rating of oral mucosal changes associated with bone marrow transplantation. *Cancer* **69**: 2469–2477

Sheridan WP, Morstyn G, Wolf M, Dodds A, Lusk J, Maher D, Layton JE, Green MD, Souza L and Fox RM (1989) Granulocyte colony stimulating factor and neutrophil recovery after high-dose chemotherapy and autologous bone marrow transplantation. *Lancet* **2**: 891–895

Sonis ST (1989) Oral complications of cancer therapy. In *Cancer: Principles and Practice of Oncology* (edn 4), DeVita VT Jr, Hellman S and Rosenberg SA. (eds), pp. 2385–2394. Lippincott: Philadelphia

Sonis ST and Costello KA (1995) A database for mucositis induced by cancer chemotherapy. *Eur J Cancer Oral Oncol* **31B**: 258–260

Sonis ST, Tracey C, Shklar G, Jensen J and Florine D (1990) An animal model for mucositis induced by cancer chemotherapy. *Oral Surg Oral Med Oral Pathol* **69**: 437–443

Sonis ST, Lindquist L, Van Vugt A, Stewart AA, Stam K, Qu GY, Iwata KK and Haley JD (1994) Prevention of chemotherapy-induced ulcerative mucositis by transforming growth factor $\beta 3^1$. *Cancer Res* **54**: 1135–1138

Spijkervet FKL, Van Saene HKF, Panders AK, Vermey A and Mehta DM (1989) Scoring irradiation mucositis in head and neck cancer patients. *J Oral Pathol Med* **18**: 161–171

Toth BB, Martin JW and Fleming TJ (1990) Oral complications associated with cancer therapy. An M.D. Anderson Cancer Center experience. *J Clin Peridontol* **17**: 508–515

Whitaker D and Williams V (1994) Cytopreparatory techniques. In *Laboratory Histopathology*, a *Complete Reference*, Woods AE and Ellis RC. (eds), pp. 10.1-1-10.1-26. Churchill Livingstone: Edinburgh

WHO (1979) *Handbook for Reporting Results of Cancer Treatment*. pp. 15–22. World Health Organization: Geneva

Woo SB, Sonis ST, Monopoli MM and Sonis AL (1993) A longitudinal study of oral ulcerative mucositis in bone marrow transplant recipients. *Cancer* **72**: 1612–1617

Wright DG, Meierovics AI and Foxley JM (1986) Assessing the delivery of neutrophils to tissues in neutropenia. *Blood* **67**: 1023–1030

© Cancer Research Campaign 1997